# Supplementary material for: 3D electron diffraction—the missing slice completing nanoscale analysis of organic solar cells in TEM
Source: Nat Commun. 2026 Apr 15;17:3159. doi: 10.1038/s41467-026-70690-y (PMC13083913; doi:10.1038/s41467-026-70690-y)
Supplement: Supplementary file 1 — Supplementary Information [file 41467_2026_70690_MOESM1_ESM.pdf]

3D Electron Diffraction – The Missing Slice Completing Nanoscale Analysis of Organic Solar Cells in TEM

Supplementary Information

Irene Kraus[1], Mingjian Wu[1], Stefanie Rechberger[1], Johannes Will[1], Santanu Maiti[2], Konstantin Dengel[1], Andreas Kuhlmann[3,4,5], Marten Huck[3,4,5], Larry Lüer[6,7], Florian Bertram[8], Hans-Georg Steinrück[3,4,5], Tobias Unruh[2], Christoph J. Brabec[6,7] and Erdmann Spiecker[1]**

[1] Institute of Micro- and Nanostructure Research & Center for Nanoanalysis and Electron Microscopy (CENEM), Friedrich-Alexander-Universität Erlangen-Nürnberg, IZNF, Cauerstraße 3, 91058 Erlangen, Germany

[2] Institute for Crystallography and Structural Physics, Friedrich-Alexander-Universität Erlangen-Nürnberg, Staudtstraße 3, 91058 Erlangen, Germany

[3] Institute for a Sustainable Hydrogen Economy (INW), Forschungszentrum Jülich GmbH, An der Deutschen Welle 7a, 52428 Jülich, Germany

[4] Institute of Physical Chemistry, RWTH Aachen University, Landoltweg 2, 52074 Aachen, Germany

[5] Department of Chemistry, Paderborn University, Warburger Straße 100, 33098 Paderborn, Germany

[6] Institute Materials for Electronics and Energy Technology (iMEET), Friedrich-Alexander-Universität Erlangen-Nürnberg, Martensstraße 7, 91058 Erlangen, Germany

[7] Helmholtz Institute Erlangen-Nürnberg for Renewable Energy (HIERN), Forschungszentrum Jülich GmbH, 91058 Erlangen, Germany

[8] Deutsches Elektronen-Synchrotron DESY, Notkestraße 85, 22607 Hamburg, Germany

*Corresponding author: mingjian.wu@fau.de, erdmann.spiecker@fau.de

The following document comprises supplementary information as referred to in the main manuscript. This includes the presented supplementary figures and tables, and a supplementary note for additional insights on STEM-EELS detection and quantification.

Supplementary Figures and Tables

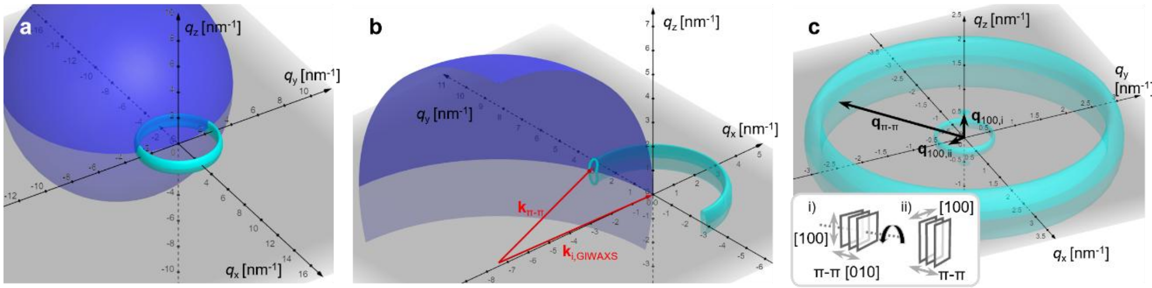

Supplementary Figure 1. GIWAXS reciprocal space sampling out of different viewing directions and more detailed reciprocal space representation of edge-on crystal orientation. a GIWAXS Ewald sphere (shown in blue) sampling of reciprocal space (coordinates q_{xyz}). **b** The curvature of the sphere has to be considered when comparing e.g. the increasing discrepancy of out-of-plane q_z axis compared to the curved sphere segment. Corresponding wavevectors **k** are shown for incident beam and π-π stacking diffraction.**c** Edge-on crystallites give rise to not only in-plane [010] π-π stacking, but also [100] lamellar stacking contributions along q_z and in-plane, depending on crystallite orientation. Scattering vectors **q** are marked accordingly.

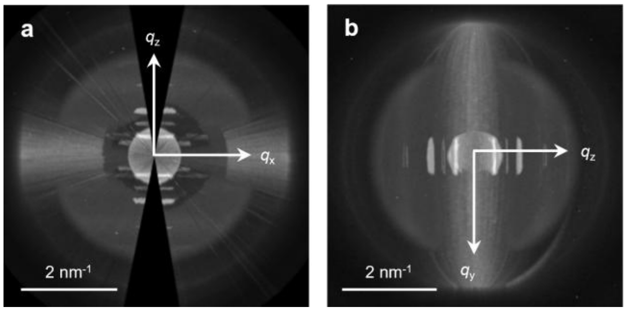

Supplementary Figure 2. Reconstructed reciprocal space volume of DRCN5T:$PC_{71}BM$ sample. This maximum intensity projection enables to visualize the inner setup of the volume. **a** With q_y reciprocal space coordinate as viewing direction the missing wedge around q_z becomes apparent. **b** The intense in-plane π-π stacking ring in the q_{xy} plane and further rings inside the volume are the most intense features which result in strong reflexes in the q_{rz} map.

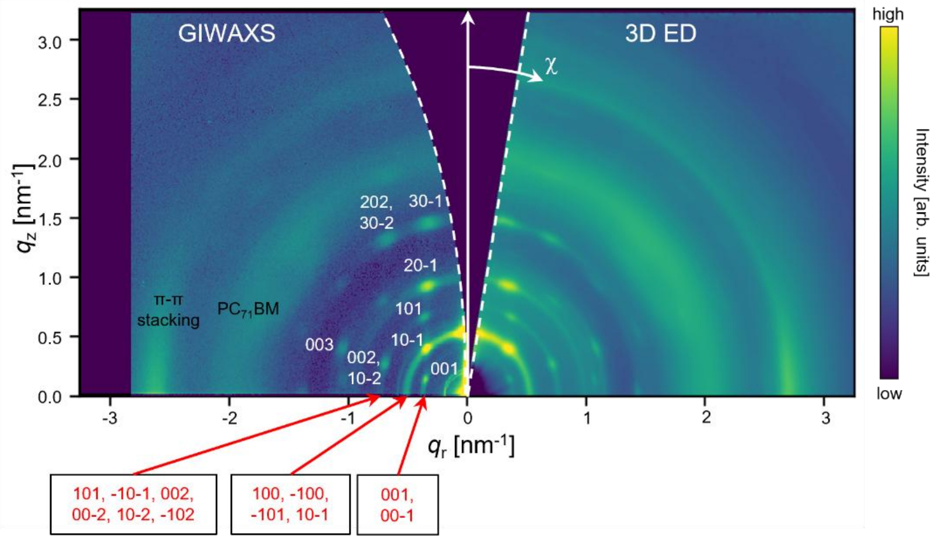

Supplementary Figure 3. Indexed *q_{rz}* maps of GIWAXS (left) and 3D ED (right). Indexing based on Berlinghof et al. [1] Reflexes indexed in red stem from face-on orientation, white labelled ones arise from edge-on orientation of the DRCN5T crystallites.. Peak width along azimuthal χ-direction represents mosaicity.

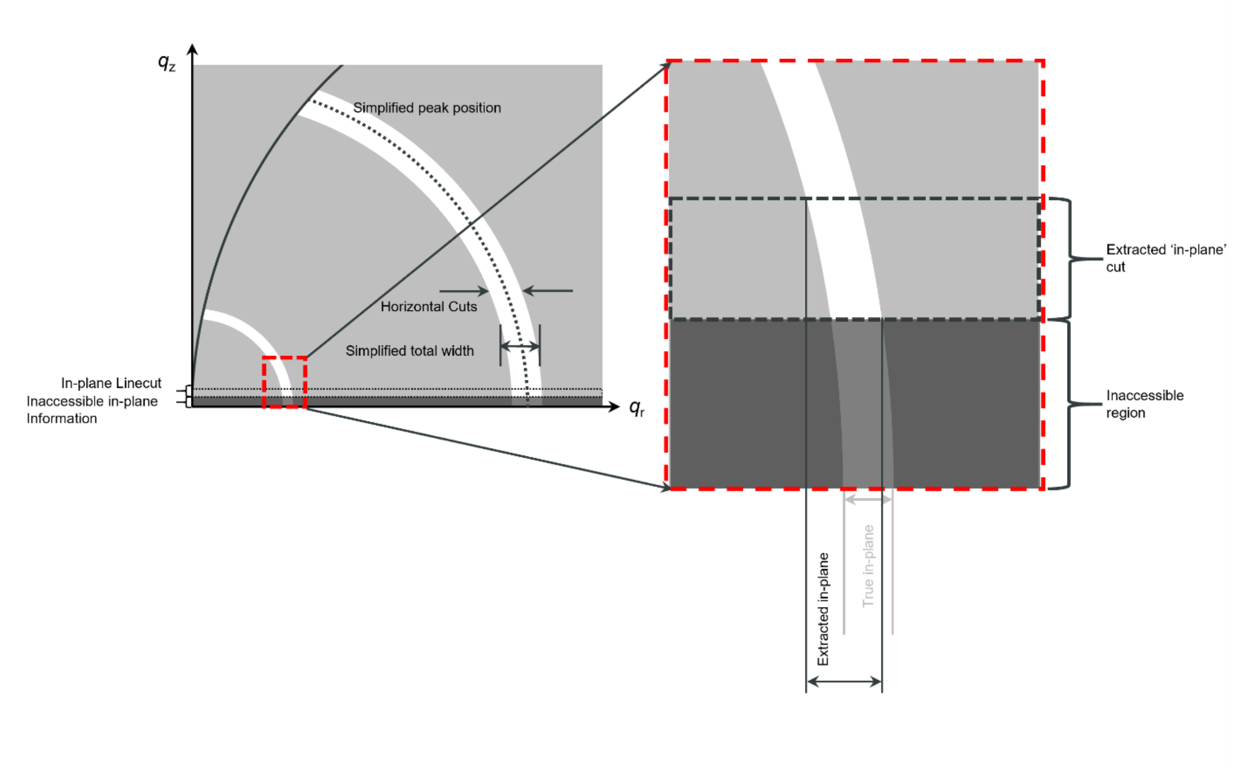

Supplementary Figure 4. GIWAXS in-plane line profile extraction displaced from true in-plane due to inaccessible region. This leads to broadening and shifting of peaks (see Supplementary Table 1). The corresponding simplifications for calculation of parameters in Supplementary Table 1 are shown. The smaller, narrower ring corresponds schematically to the (100) ring at 0.53 nm^{-1}, the broader ring with larger radius to the π-π stacking ring at 2.73 nm^{-1}. We note that extraction of linecuts in q_z-χ maps (instead of q_{rz} as shown here) could circumvent broadening due to ring curvature; furthermore, in case of diffraction rods also the broadening and shifting is not an issue. In both cases the in-plane region would still be shadowed due to finite incidence angles on the sample.

Supplementary Table 1. Peak positions and widths for (100) and π-π ring calculated for in-plane data extraction displaced from true in-plane (IP).

	q_z cut position [nm^{-1}]	q_r- position π-π [nm^{-1}]	Cut width [nm^{-1}]	Relative peak position difference to IP [%]	Relative peak width difference to IP [%]
π-π ring	0 (IP)	2.73	0.2	0	0
	0.01	2.729982	0.200001	0.000671	0.000671
	0.02	2.729927	0.200005	0.002684	0.002687
	0.03	2.729835	0.200012	0.006038	0.006046
	0.04	2.729707	0.200022	0.010735	0.010750
	0.05	2.729542	0.200034	0.016773	0.016799
	0.06	2.729341	0.200048	0.024155	0.024193
	$q_{z,cut}$ range	Mean Position	Total width		
	0.01-0.06	2.729722	0.200666	0.0101759	0.3334159
(100) ring	0 (IP)	0.53	0.05	0	0
	0.01	0.529906	0.050009	0.017802	0.017844
	0.02	0.529623	0.050036	0.071225	0.071435
	0.03	0.529150	0.050080	0.160328	0.160945
	0.04	0.528488	0.050143	0.285206	0.286666
	0.05	0.527636	0.050225	0.445993	0.449006
	0.06	0.526593	0.050324	0.642864	0.648499
	$q_{z,cut}$ range	Mean Position	Total width		
	0.01-0.06	0.528566	0.053487	0.270569	6.973855

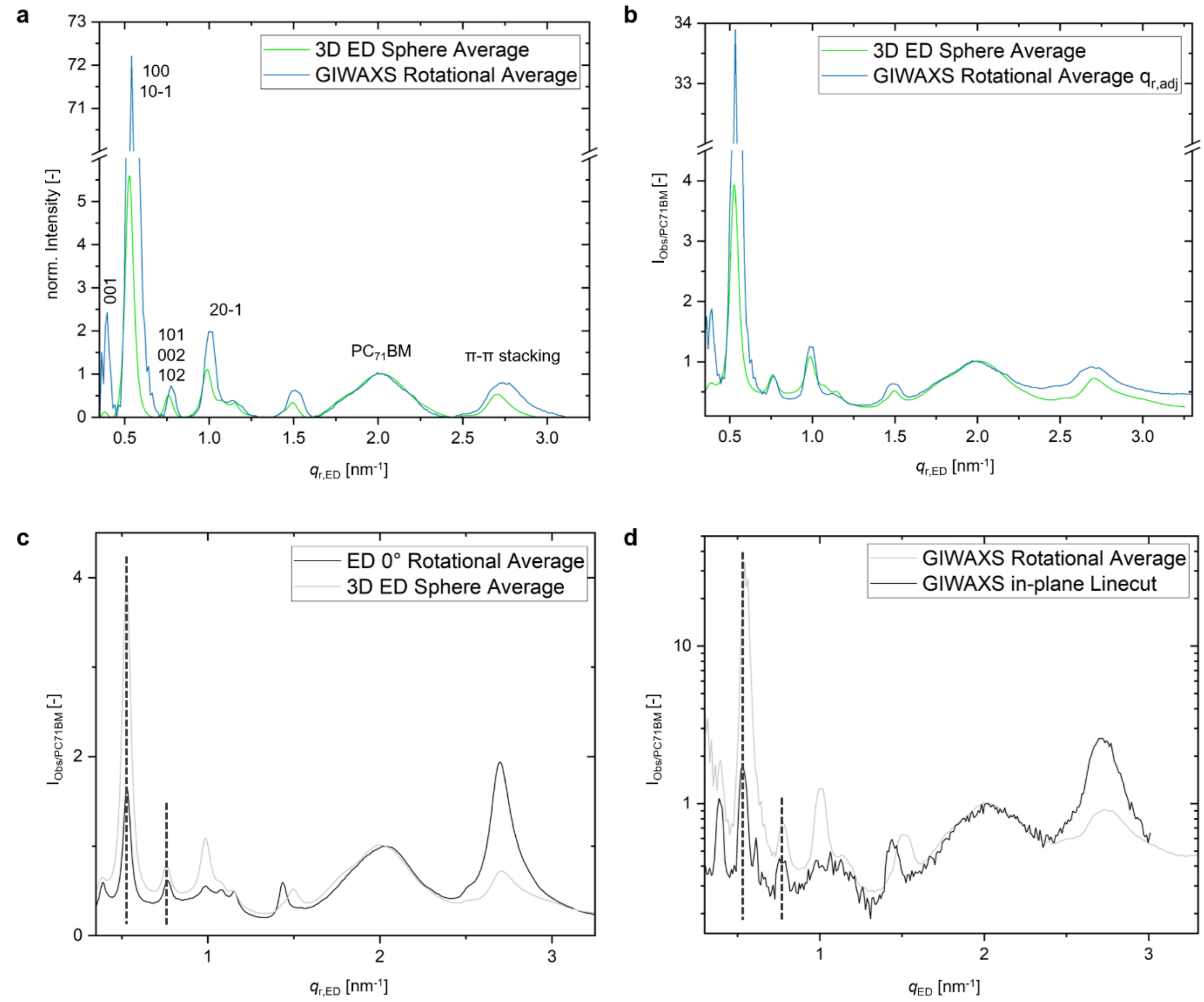

Supplementary Figure 5. Additional plots for clarification of the GIWAXS rotational average shift. **a** The q_r scale of the GIWAXS data in the main article was adjusted by 1.5%, here an unadjusted version of the averages of 3D ED and GIWAXS is shown. **b** The adjusted dataset shows good agreement with 3D ED as shown here in the profiles normalized to the $PC_{71}BM$ peak without background subtraction. **c** The ED 0° profile and the 3D ED sphere average match well, **d** while the GIWAXS in-plane profile and the corresponding rotational average feature a shift relative to each other due to refraction and reflection effects.

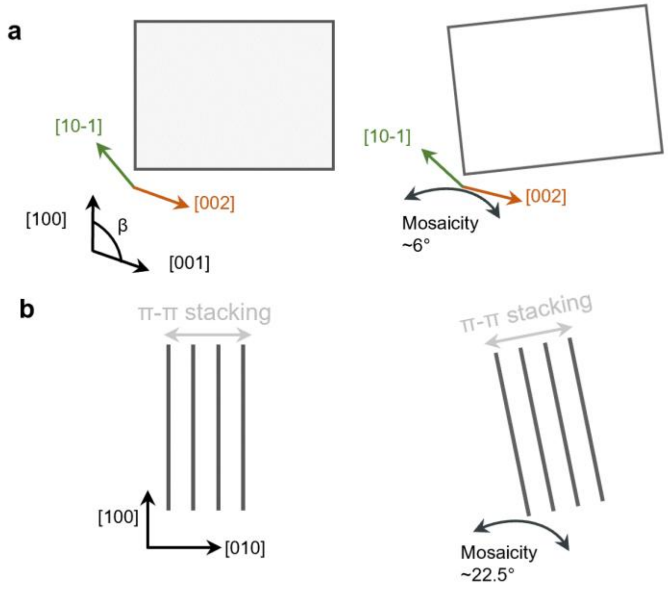

Supplementary Figure 6. Mosaicity of DRCN5T crystallites. **a** The mosaicity of [10-1] and [002] should be related based on geometric considerations of the unit cell, as the mosaicity is related to an inclination around the b-axis pointing into the screen. **b** The π-π stacking mosaicity is referring to a different rotation axis of the crystallites, namely the c-axis, and can therefore have a different value. Based on unit cell as determined by Berlinghof et al.[1]

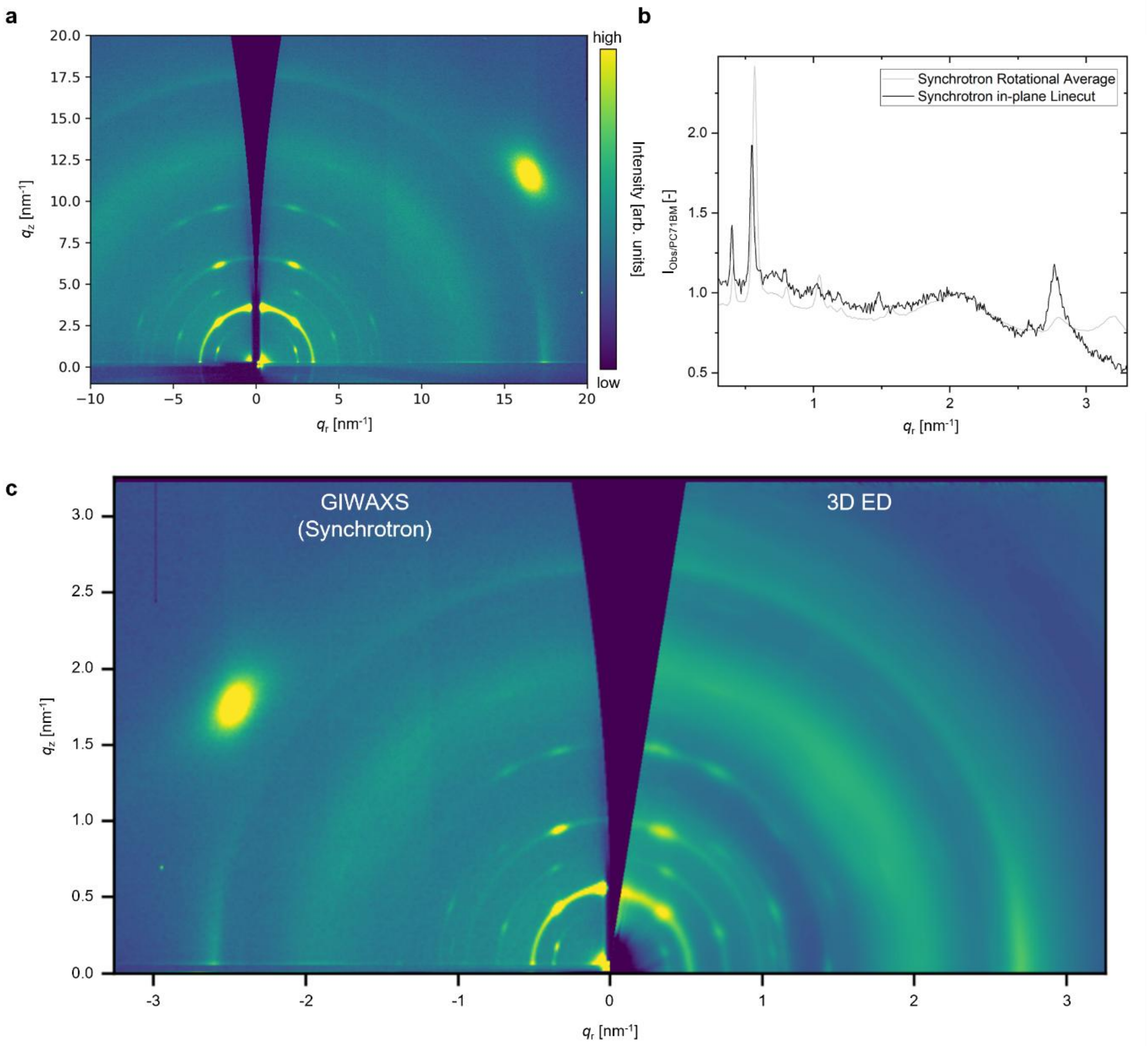

Supplementary Figure 7. GIWAXS synchrotron data. a q_{rz} map of the synchrotron measurement with q-scaling in GIWAXS notation. **b** Extracted in-plane line profile and rotational average show a shift of the rotational average to larger q values (ED notation for q). **c** The qualitative comparison of the synchrotron GIWAXS map and 3D ED show good agreement (ED notation).

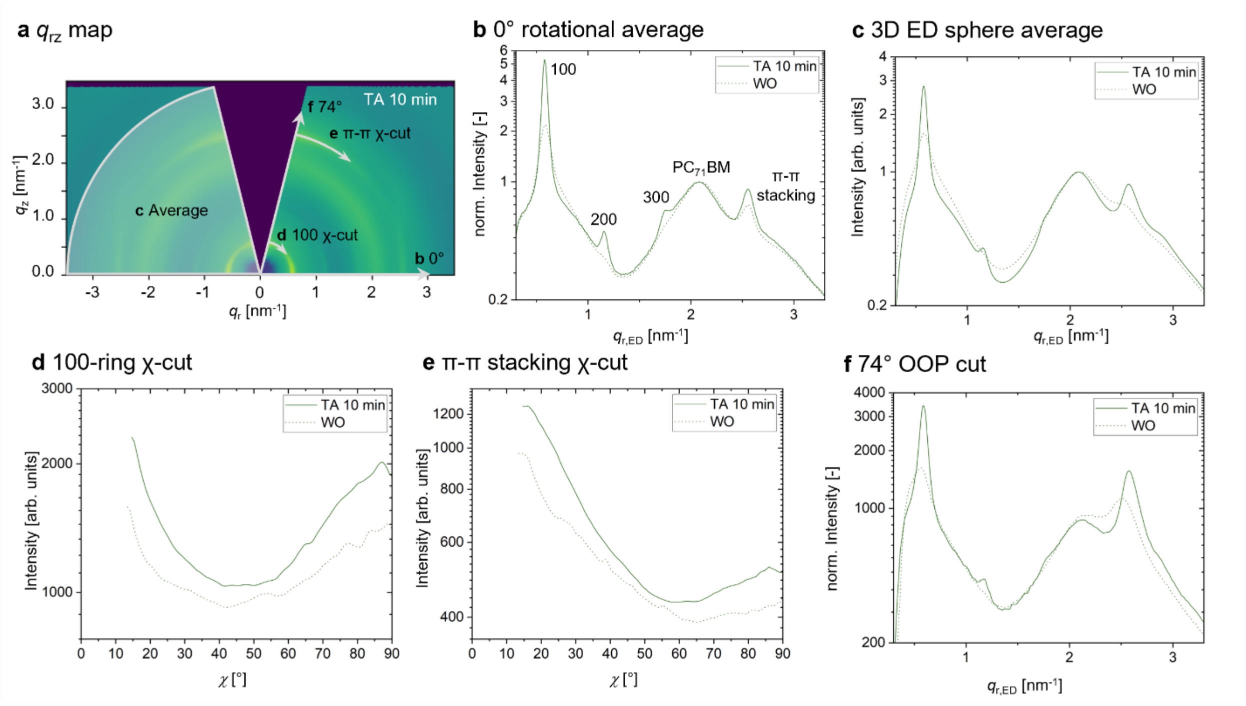

Supplementary Figure 8. Intensity profiles extracted from q_{rz} maps for P3HT:PC$_{71}$BM samples (WO and TA 10 min). **a** q_{rz} map of P3HT:PC$_{71}$BM thermally annealed for 10min with schematics representing extraction of the following intensity profiles: **b** 0° (in-plane) rotational averages (data based on 0° diffraction patterns), **c** 3D ED sphere averages, , (incorporating full map intensity), **d** χ-cut of 100 ring and **e** of π-π stacking ring, and **f** 74° (out-of-plane) cuts.

Supplementary Table 2. CCL of P3HT:PC$_{71}$BM system for WO and TA 10min sample. (100) and π-π stacking peak were analyzed in-plane and out-of-plane (74° cut relative to q_r axis in 3D ED q_{rz} map).

CCL [nm]	WO	TA 10min
In-plane		
(100)	8.40±0.09	16.01±0.07
π-π stacking	7.85±0.11	8.50±0.08
Out-of-plane		
(100)	8.50±0.25	11.62±0.25
π-π stacking	4.90±0.06	5.69±0.12

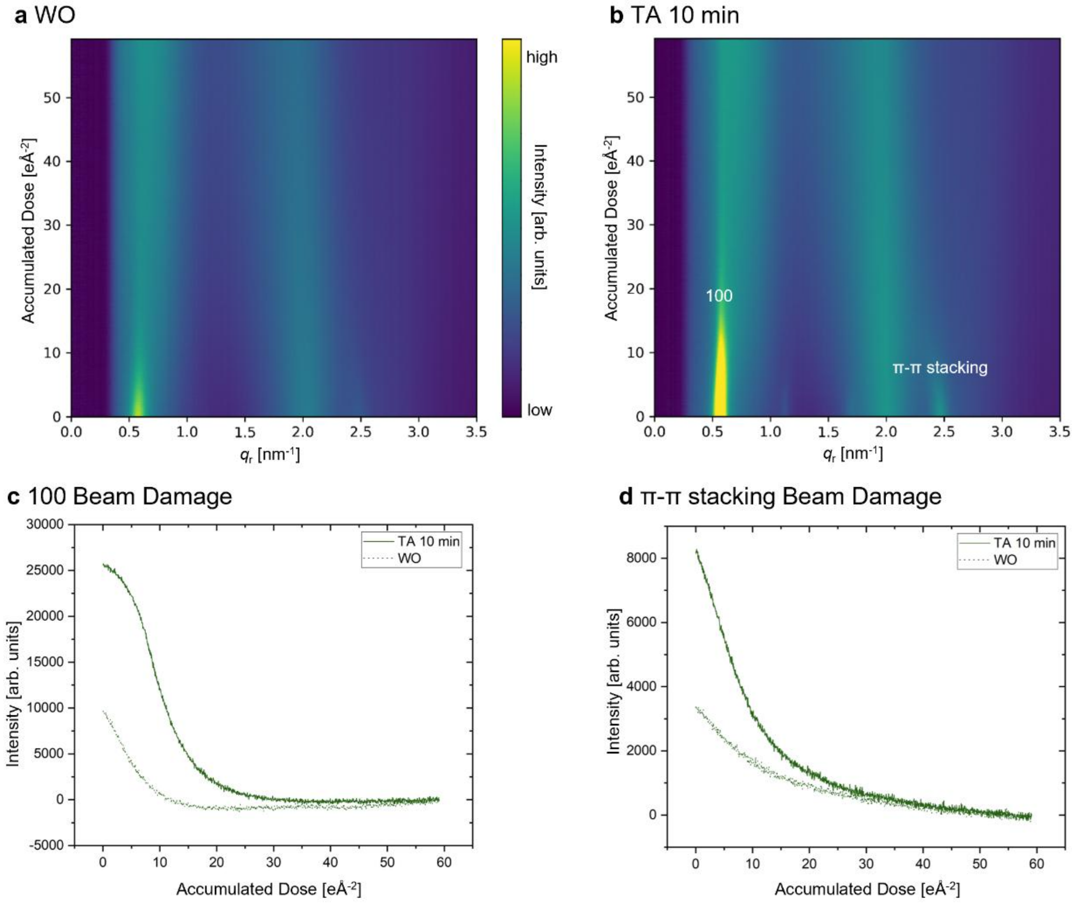

Supplementary Figure 9. Beam damage behavior of P3HT:PC_{71}BM system WO and TA 10min under the electron beam. **a,b** A series of diffraction patterns was acquired at one position, capturing the degradation behavior. The diffraction patterns were polar transformed and projected along azimuth axis, resulting in a *q*-*t* (i.e., *q*-dose) map depicting diffraction intensity as function of accumulated dose. **c,d** Linecuts of 100 and π-π stacking peak were extracted along accumulated dose, showing the decreasing intensity of diffraction peaks.

Supplementary Table 3. Critical dose D_c of WO and TA 10min P3HT:PC_{71}BM system. The values are based on the intensity profiles in Supplementary Figure 9c,d, by determining the accumulated dose value that reduces the initial intensity by a factor of 1/e.

D_c [e^-Å^{-2}]	WO	TA 10min
(100)	5.3	11.4
π-π stacking	13.2	10.6

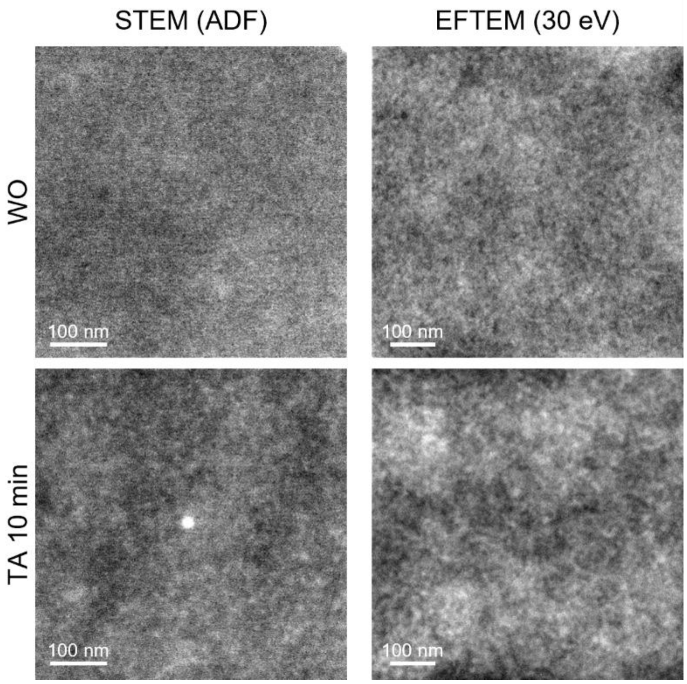

Supplementary Figure 10. STEM and EFTEM images of P3HT:$PC_{71}BM$ WO and TA 10min sample. EFTEM images use the plasmon signal of $PC_{71}BM$ at 30 eV, i.e. $PC_{71}BM$ rich regions appear bright, P3HT rich regions darker. The STEM images show coarsening of nanomorphology and EFTEM reveals increased phase separation upon thermal annealing.

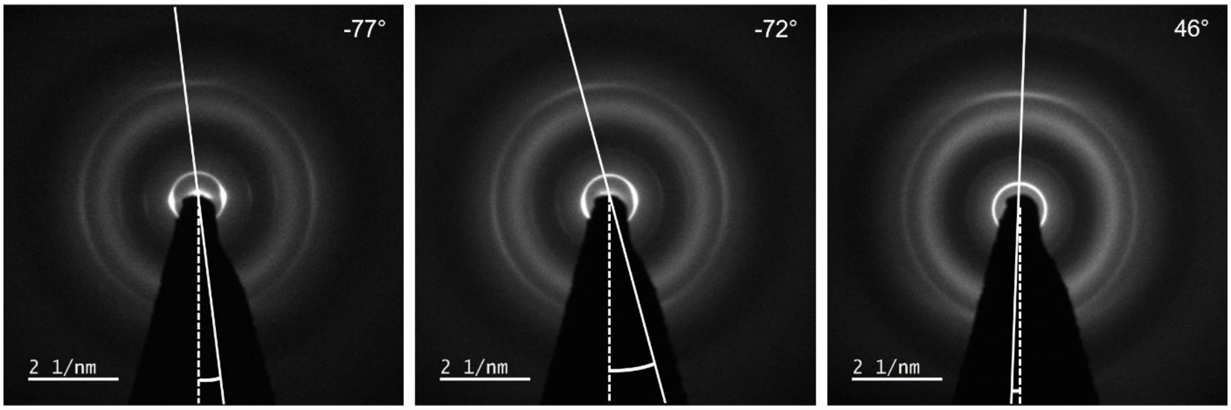

Supplementary Figure 11. Influence of film buckling on recorded diffraction patterns. In case the diffraction geometry is not consistent due to buckling of the film on the TEM grid, this is represented in the recorded diffraction patterns. In the shown patterns of the TA P3HT:$PC_{71}BM$ system, acquired at different nominal tilt angles, this results in a varying angular position of the in-plane π-stacking peak as marked by the white filled lines relative to the dashed line representing the image horizontal. A possible data processing approach to minimize the influence on q_{rz} map reconstruction is to rotate the diffraction patterns for the tilt axes to align.

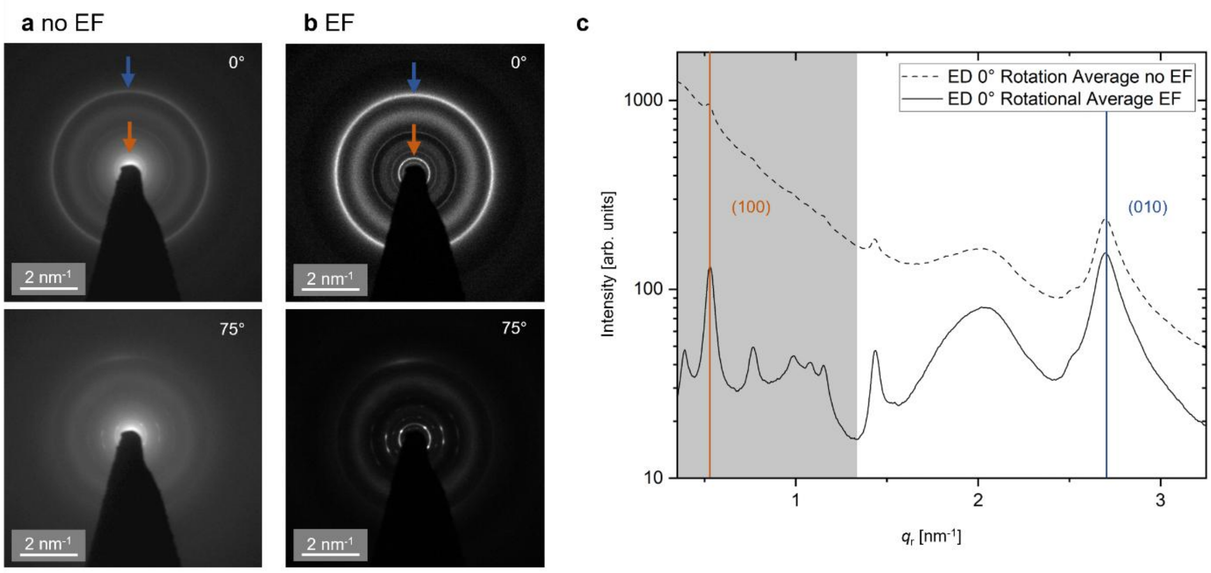

Supplementary Figure 12. Effect of energy filtering on ED data. Diffraction patterns acquired **a** without a 10 eV energy-filter slit (intensity scaled for better visibility and comparability) and **b** with energy filtering from the same DRCN5T:$PC_{71}BM$ film at 0° and 75° tilt, and **c** line profiles comparing the two conditions. The unfiltered data show a significantly higher background at low scattering vectors (shaded region) due to inelastic electrons, which obscures weaker diffraction peaks. With elastic filtering, the background is markedly reduced, allowing clear detection of the (100) lamellar and (010) π–π stacking reflections (markings). This highlights the importance of energy filtering for accurate 3D ED of organic thin films.

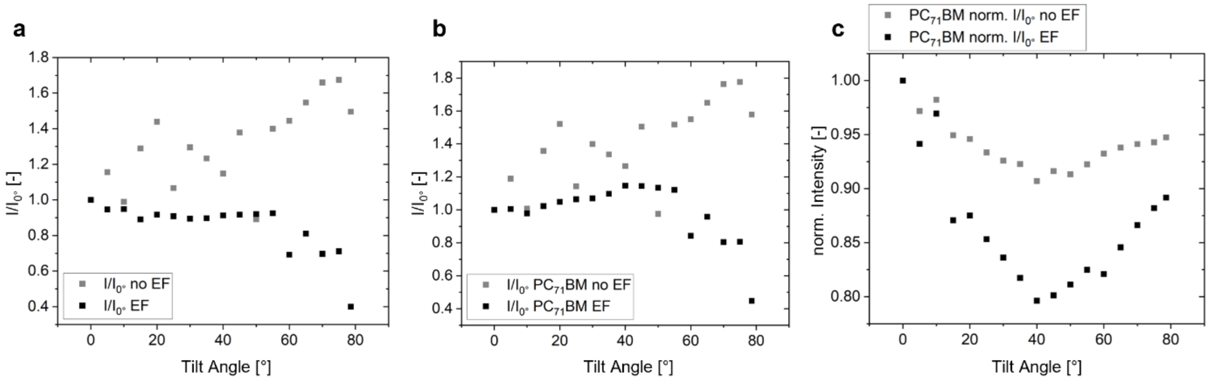

Supplementary Figure 13. Evaluation of the diffracted intensity (I) with respect to the intensity at 0 degree (I_0) as function of sample tilt, with and without energy filtering (EF, 10 eV). **a** Diffraction intensity of the whole diffraction pattern (excluding the beam-stopper) relative to the 0° pattern intensity. To exclude an influence of the direct beam position relative to the beam-stopper, a radial range of 0.67-3.42 nm^{-1} is chosen. **b** Diffraction intensity in the radial range of $PC_{71}BM$, i.e., 1.66-2.37 nm^{-1} relative to the 0° pattern intensity. **c** Assuming $PC_{71}BM$ is isotropic (no texture, i.e., preferred orientation), a normalization factor of the diffracted intensity of the whole pattern is derived (from data points in b), and used to account for the different beam-specimen interaction volume as function of tilt.

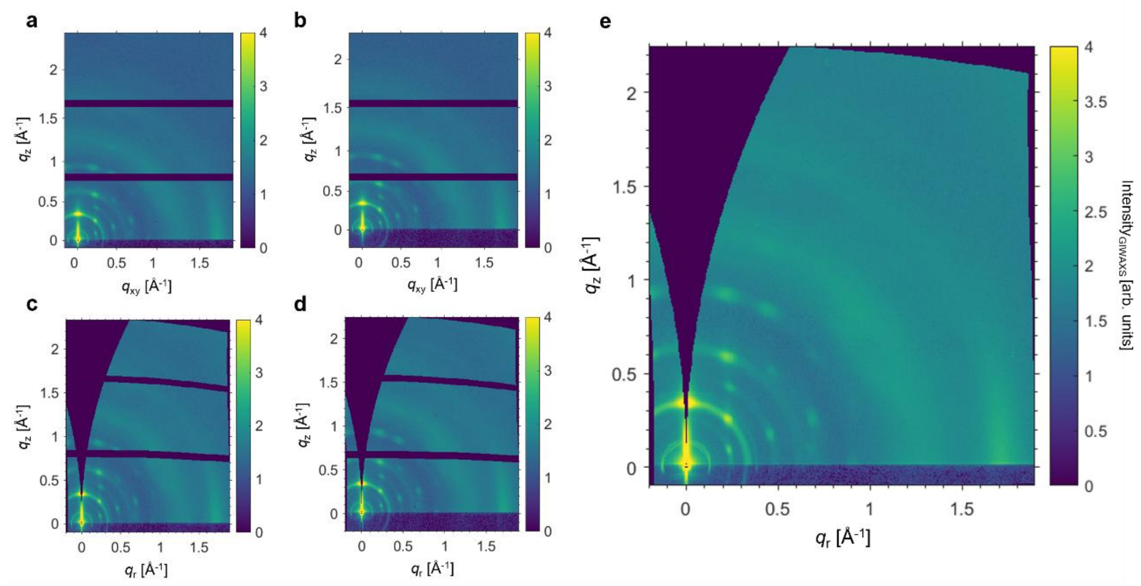

Supplementary Figure 14. Adding of two GIWAXS measurements and coordinate transformation. All patterns shown in *q*-scaling of GIWAXS notation. **a,b** By adding two GIWAXS datasets acquired with different detector positions, missing segments due to the detector setup can be compensated. **c,d** By coordinate transformation to take into account the Ewald sphere reciprocal space sampling, the missing wedge along q_z becomes visible, **e** also for the pattern comprised of the two single measurements.

Supplementary Note: STEM-EELS detection and quantification

The STEM-EELS data is depicted in Fig. 5 in the main manuscript. The C K signal shows the carbon richer $PC_{71}BM$ as bright whereas the S L signal results in the DRCN5T regions being depicted as bright due to the higher sulfur content of the small molecule. The small molecule shows leaf like structures. Additionally, single pixel spectra from different sample regions are shown. The S L-edge map was extracted by fitting the signal from ~110–410 eV using the model-based fitting algorithm as implemented in GMS[2], which yields a discernible map due to the optimized acquisition conditions used. Note, that the O K-edge (~532 eV) and N K-edge (~400 eV) signals were not detected in our EELS dataset because the acquisition parameters were optimized for the C-K and S-L edges and the limited CCD detector's efficiency and high noise. In a separate test on a similar OSC sample, a direct electron detector was able to capture the N K-edge under comparable conditions.[3]

Supplementary References

1 Berlinghof, M., Langner, S., Harreiß, C., Schmidt, E. M., Siris, R., Bertram, F., Shen, C., Will, J., Schindler, T., Prihoda, A., Rechberger, S., Duesberg, G. S., Neder, R. B., Spiecker, E., Brabec, C. J. & Unruh, T. Crystal-structure of active layers of small molecule organic photovoltaics before and after solvent vapor annealing. *Zeitschrift für Kristallographie - Crystalline Materials* **235**, 15–28 (2020). https://doi.org/10.1515/zkri-2019-0055

2 Verbeeck, J. & Van Aert, S. Model based quantification of EELS spectra. *Ultramicroscopy* **101**, 207-224 (2004). https://doi.org/10.1016/j.ultramic.2004.06.004

3 Fürk, P., Mallick, S., Rath, T., Reinfelds, M., Wu, M., Spiecker, E., Simic, N., Haberfehlner, G., Kothleitner, G., Ressel, B., Holler, S., Schaubeder, J. B., Materna, P., Amenitsch, H. & Trimmel, G. The challenge with high permittivity acceptors in organic solar cells: a case study with Y-series derivatives. *Journal of Materials Chemistry C* **11**, 8393–8404 (2023). https://doi.org/10.1039/d3tc01112g
