# Supplementary material for: 3D electron diffraction—the missing slice completing nanoscale analysis of organic solar cells in TEM
Source: Nat Commun. 2026 Apr 15;17:3159. doi: 10.1038/s41467-026-70690-y (PMC13083913; doi:10.1038/s41467-026-70690-y)
Supplement: Supplementary file 2 — Description of Additional Supplementary Files [file 41467_2026_70690_MOESM2_ESM.pdf]

Description of Additional Supplementary Files:

Supplementary Movie 1: Diffraction patterns of the DRCN5T:PC71BM tilt series with 1° step size. The patterns are sorted from -78° to 80°, shifted to one common center, rotated for the tilt axis to coincide with the vertical image axis and the intensity is normalized with respect to the PC71BM ring.

Supplementary Movie 2: 3D ED Volume of DRCN5T:PC71BM sample in maximum intensity projection representation rotating around q_z axis.

Supplementary Movie 3: 3D ED Volume of DRCN5T:PC71BM sample in maximum intensity projection representation rotating around q_y axis.
